# miR-29a-3p/THBS2 Axis Regulates PAH-Induced Cardiac Fibrosis

**DOI:** 10.3390/ijms221910574

**Published:** 2021-09-30

**Authors:** Chih-Hsin Hsu, I-Fan Liu, Hsuan-Fu Kuo, Chia-Yang Li, Wei-Shiung Lian, Chia-Yuan Chang, Yung-Hsiang Chen, Wei-Lun Liu, Chi-Yu Lu, Yu-Ru Liu, Tzu-Chieh Lin, Tsung-Ying Lee, Chi-Yuan Huang, Chong-Chao Hsieh, Po-Len Liu

**Affiliations:** 1 Department of Internal Medicine, National Cheng Kung University Hospital, College of Medicine, National Cheng Kung University, Tainan 704, Taiwan; chihhsinhsu@gmail.com; 2 Institute of Clinical Medicine, National Yang Ming Chiao Tung University, Taipei 112, Taiwan; Wes0208@yahoo.com.tw; 3 Heart Center, Cheng Hsin General Hospital, Taipei 112, Taiwan; 4 Graduate Institute of Medicine, College of Medicine, Kaohsiung Medical University, Kaohsiung 807, Taiwan; medsnail@hotmail.com (H.-F.K.); chiayangli@kmu.edu.tw (C.-Y.L.); 990327kmuh@gmail.com (T.-C.L.); 5 Department of Internal Medicine, Kaohsiung Municipal Ta-Tung Hospital, Kaohsiung Medical University Hospital, Kaohsiung Medical University, Kaohsiung 807, Taiwan; 6 Department of Internal Medicine, School of Medicine, College of Medicine, Kaohsiung Medical University, Kaohsiung 807, Taiwan; 7 Division of Cardiology, Department of Internal Medicine, Kaohsiung Medical University Hospital, Kaohsiung Medical University, Kaohsiung 807, Taiwan; 8 Core Laboratory for Phenomics and Diagnostic, Kaohsiung Chang Gung Memorial Hospital, Kaohsiung 833, Taiwan; lianws@gmail.com; 9 Department of Medical Research, Kaohsiung Chang Gung Memorial Hospital, Kaohsiung 833, Taiwan; 10 Department of Mechanical Engineering, National Cheng Kung University, Tainan 701, Taiwan; cychang0829@gmail.com; 11 Graduate Institute of Integrated Medicine, College of Chinese Medicine, China Medical University, Taichung 404, Taiwan; yhchen@mail.cmu.edu.tw; 12 Department of Psychology, College of Medical and Health Science, Asia University, Taichung 413, Taiwan; 13 School of Medicine, College of Medicine, Fu Jen Catholic University, New Taipei City 242, Taiwan; medrpeterliu@gmail.com; 14 Division of Critical Care Medicine, Department of Emergency and Critical Care Medicine, Fu Jen Catholic University Hospital, Fu Jen Catholic University, New Taipei City 243, Taiwan; 15 Department of Biochemistry, College of Medicine, Kaohsiung Medical University, Kaohsiung 807, Taiwan; cylu@kmu.edu.tw; 16 Department of Respiratory Therapy, College of Medicine, Kaohsiung Medical University, Kaohsiung 807, Taiwan; lu6525@ms42.hinet.net (Y.-R.L.); ja321cky@gmail.com (T.-Y.L.); sh12010929@gmail.com (C.-Y.H.); 17 Graduate Institute of Clinical Medicine, College of Medicine, Kaohsiung Medical University, Kaohsiung 807, Taiwan; 18 Division of Cardiovascular Surgery, Department of Surgery, Kaohsiung Medical University Hospital, Kaohsiung Medical University, Kaohsiung 807, Taiwan; 19 Department of Surgery, Faculty of Medicine, College of Medicine, Kaohsiung Medical University, Kaohsiung 807, Taiwan; 20 Department of Medical Research, Kaohsiung Medical University Hospital, Kaohsiung 807, Taiwan

**Keywords:** miR-29a-3p, THBS2, cardiomyocytes, fibrosis, pulmonary arterial hypertension

## Abstract

Pulmonary artery hypertension (PAH) pathology involves extracellular matrix (ECM) remodeling in cardiac tissues, thus promoting cardiac fibrosis progression. miR-29a-3p reportedly inhibits lung progression and liver fibrosis by regulating ECM protein expression; however, its role in PAH-induced fibrosis remains unclear. In this study, we aimed to investigate the role of miR-29a-3p in cardiac fibrosis progression in PAH and its influence on ECM protein thrombospondin-2 (THBS2) expression. The diagnostic and prognostic values of miR-29a-3p and THBS2 in PAH were evaluated. The expressions and effects of miR-29a-3p and THBS2 were assessed in cell culture, monocrotaline-induced PAH mouse model, and patients with PAH. The levels of circulating miR-29a-3p and THBS2 in patients and mice with PAH decreased and increased, respectively. miR-29a-3p directly targets THBS2 and regulates THBS2 expression via a direct anti-fibrotic effect on PAH-induced cardiac fibrosis. The circulating levels of miR-29a-3p and THBS2 were correlated with PAH diagnostic parameters, suggesting their independent prognostic value. miR-29a-3p targeted THBS2 expression via a direct anti-fibrotic effect on PAH-induced cardiac fibrosis, indicating miR-29a-3p acts as a messenger with promising therapeutic effects.

## 1. Introduction

Pulmonary arterial hypertension (PAH) is an incurable disease resulting in the death of the patients due to right ventricular failure [1,2]; it is characterized by extracellular matrix (ECM) remodeling of the pulmonary arteries with increased collagen deposition, collagen cross-linkage, and elastic laminae breakdown. ECM remodeling frequently occurs during the early stage of PAH and plays a critical role in the pathogenesis of distal pulmonary vascular remodeling [3,4,5]. In addition, the ECM network plays a crucial role in cardiac homeostasis by providing structural support, facilitating force transmission, and transducing key signals to cardiomyocytes, vascular cells, and interstitial cells [6]. Matricellular proteins are upregulated in the injured and remodeling heart and play an essential role in regulating inflammatory, reparative, fibrotic, and angiogenic pathways. Although matricellular proteins do not play a primary role in tissue architecture, they are induced following injury and are critical in modulating cell–cell and cell–matrix interactions [6]. When released into the matrix, matricellular proteins associate with growth factors, cytokines, and other bioactive effectors and bind to cell-surface receptors that subsequently regulate the signal-transduction cascades [7].

Thrombospondin 2 (THBS2) is an anti-angiogenic matricellular protein that is an essential modulator of ECM homeostasis in fibrotic progression [8,9]. THBS2 is a stromal cell and an exosomal protein that cells release into the surrounding microenvironment or circulation to affect the entire body. THBS2 is highly expressed in cardiac hypertrophy, dilative cardiomyopathy [10], fibrosclerotic and stenotic aortic valves [11], pressure-overloaded heart, and aging heart [12]. High levels of circulating thrombospondin 1 (THBS1) and THBS2 are correlated with the PAH [13] and poor prognosis of patients with heart failure [14]. THBS2 protects the pressure-overloaded heart from rupture and aging heart from dilative cardiomyopathy [10] as well as protects cardiomyocytes and prevents matrix disruption in doxorubicin-induced cardiomyopathy [15]. Thus, THBS2 plays an essential role in the pathological process in cardiac tissues [16,17]. Idiopathic PAH (IPAH) reportedly increases the expression of ECM genes, such as collagen type III, α-1, tenascin C, collagen type VI α-3, and von Willebrand factor, including THBS2 [18]. However, the function and role of THBS2 in the progression of PAH remain unclear.

Recent studies suggest that cell-specific, cardiomyocyte-derived exosomal microRNAs (miRNAs) and proteins have distinct pathophysiological functions in cells located in the cardiac microenvironment, including fibroblasts, cardiomyocytes, endothelial cells, and mesenchymal stem cells [19]. In cardiac fibroblasts, cardiomyocyte-derived exosomes regulate the expression of genes related to cardiac fibrosis [20]. In cardiomyocytes, cardiomyocyte-derived exosomes regulate apoptosis, autophagy, and inflammation [21,22]. In endothelial cells, cardiomyocyte-derived exosomes regulate angiogenesis and glucose transport, and metabolism [23]. In bone marrow-derived mesenchymal stem cells, cardiomyocyte-derived exosomes regulate autophagy and apoptosis [24,25]. Very few studies have identified circulating miRNAs in peripheral venous blood of patients with PAH and those associated with cardiac fibrosis, hypertrophy, heart failure, and survival [26,27]. miR-29a-3p is known to target transcripts of collagens and various ECM proteins [28], with the downregulation of miR-29 contributing to the development of systemic sclerosis [29] as well as pulmonary, kidney, and liver fibrosis [30,31,32]. Upregulating miR-29 presents a promising anti-fibrotic therapy for lung, liver, kidney, and heart diseases [33,34]. Both hypertensive right ventricle and pulmonary artery act as “endocrine or paracrine initial organs”, secreting exosomal miRNAs into the circulation that can be taken up by distant cells causing epigenetic regulation of the recipient cells. The circulating miR-29a-3p levels in patients with PAH have not been investigated to date. In the present study, we aimed to investigate the level of miR-29a-3p in circulation and its role in PAH-related cardiac fibrosis. Moreover, the regulatory effects of miR-29a-3p and THBS2 in PAH-induced cardiac fibrosis were examined.

## 2. Materials and Methods

### 2.1. Patients and Hemodynamic Analysis

We enrolled 21 patients with PAH (Table S1) who were admitted to the National Cheng Kung University Hospital for pulmonary artery catheterization and 21 healthy donors (NR) (Table S2). We performed right heart catheterization, considered the gold standard for the diagnosis and follow-up of PAH, and recorded the pressure in the right atrium as well as right ventricular and pulmonary arteries during the procedure. Cardiac output was measured by the thermodilution technique. We prospectively collected peripheral, right atrial, right ventricular, and main pulmonary arterial blood samples separately during right heart catheterization. Blood samples were collected in EDTA tubes, centrifuged at 3000  rpm for 15  min at 4 °C, and the obtained sera were stored at −80 °C. Plasma was separated by centrifugation at 1500× *g* for 10 min at 4 °C and stored until further use. Each patient provided informed consent. This study was conducted according to the recommendations of the 1975 Declaration of Helsinki on Biomedical Research involving human subjects and was approved by the local ethics committee of the National Cheng-Kung University Hospital (IRB: B-ER-106-056).

### 2.2. Echocardiography

Standard echocardiography was performed in patients using Philips iE33 Ultrasound Machine (Philips, Tampa, FL, USA) with a 3.5-MHz multiphase-array probe in accordance with the recommendations of the American Society of Echocardiography [35]. The tricuspid valve was investigated for the presence of tricuspid regurgitation with color and continuous-wave Doppler in the apical 4-chamber view. The transducer was aligned to achieve the maximal peak velocity on tricuspid regurgitation. Based on the trans-tricuspid flow velocity, RV pressure was calculated by applying the Bernoulli equation. Tricuspid annular plane systolic excursion (TAPSE) represents the displacement of the RV annular plane toward the apex during systole. All measurements were performed by an experienced sonographer (CHH) who was blinded to the treatment groups.

### 2.3. Monocrotaline (MCT)-Induced PAH Animal Model

This experiment was approved by the Institutional Animal Care Committee of Kaohsiung Medical University (license number: 108265), and the experimental facility is accredited by the International Association for the Evaluation and Accreditation of Laboratory Animal Care (AAALAC). Eight-week-old male C57BL/6 mice were purchased from BioLASCO Taiwan Co., Ltd. (Taipei, Taiwan). Mice were divided into two groups and received either a single subcutaneous injection of MCT (60 mg/kg; *n* = 6; MCT-treated group) or an equal volume of phosphate buffer solution (PBS; *n* = 6; control group). After 30 days, the mice were euthanized with tiletamine-zolazepam (Zoletil 50; 10 mg/kg intraperitoneal injection) and 1 mL of blood from the left ventricle, and the whole hearts were immediately collected for further analyses. All mice were weighed weekly during the experimental period, and weight changes were closely observed. If the weight was reduced by ≥20% of the original body weight, the animal was excluded from the study.

### 2.4. Micro-Computed Tomography (MicroCT)

MicroCT analyses were performed as described previously [36]. At the end of the in vivo experiment (day 30), each mouse was adequately anesthetized using isoflurane and settled with tape in a supine position on the surgery platform for whole-body reperfusion. Samples were scanned using a desktop microCT (SkyScan 1172 or 1272, Bruker MicroCT, Kontich, Belgium). MicroCT projections were back projection-reconstructed using the NRecon software (NreconServer64bit, Bruker MicroCT) and volume-rendered and visualized in 3D using the CTVox software (Bruker MicroCT). Muscle tissues and blood vessels were segmented and analyzed using the CTAn software (Bruker MicroCT). Blood vessel sizes were assessed using Matlab (The MathWorks, Inc., Natick, MA, USA) and plotted using Excel (Microsoft Corporation, Redmond, WA, USA). The experimental animals were euthanized and set onto the dissecting tray for perfusion, and their hearts were injected with a silicone rubber compound (Microfil^®^ MV-122, yellow, Flow-Tech, Carver, MA, USA). All steps were performed according to the detailed instructions provided in the operation procedures [36]. The microCT scans were evaluated using SkyScan 1176 microCT system with 50 kV/500 μA X-ray working energy and 9 μm resolution for cardiovascular visualization. The CTAn software was capable of 3D reconstructions and graphic visualization of the vascular distribution.

### 2.5. Cell Culture

Primary human cardiac fibroblasts (HCF; C-12375; PromoCell, Heidelberg, Germany) and primary human cardiac myocytes (HCM; ScienCell Research Laboratories, Carlsbad, CA, USA) were isolated from the ventricles of an adult heart and cultured in a specific culture media, namely, HCF growth medium (C-23010; PromoCell) and HCM medium (ScienCell Research Laboratories) supplemented with 1% penicillin/streptomycin solution (ScienCell Research Laboratories, Carlsbad, CA, USA) and 5% fetal bovine serum. Cells were cultured in a cell culture incubator in 5% CO_2_ at 37 °C and then subcultured and sub-distributed at 80% confluency. The culture medium was replaced every 3–4 days. Cells past the ninth subculture generation were not used.

### 2.6. Masson’s Trichrome Staining and Immunostaining

To investigate the morphological changes of PAH-induced cardiac fibrosis, 5-μm-thick heart tissue sections were examined by hematoxylin and eosin stain (Thermo Fisher Scientific, Waltham, MS, USA) and Masson’s trichrome staining kit (Invitrogen, Carlsbad, CA, USA). Different myocardial tissues were stained in different colors: cardiac muscle fibers (red color), collagen or fibrosis area (blue color), cytoplasm (light red or pink color), and cell nuclei (dark brown or black color). To detect the uptake of exosomes by cardiac fibroblasts, the exosomes were labeled with 1,1′-dioctadecyl-3,3,3′,3′-tetramethylindocarbocyanine perchlorate (Dil) fluorescent dye (Thermo Fisher Scientific) and incubated with HCFs, and then these cells were examined under a confocal laser microscope (Leica, Baca Raton, FL, USA). To detect the expression of exosomal marker proteins, early endosome antigen 1 (EEA1), and myofibroblast marker proteins, HCFs or heart tissues were treated with exosomes or recombinant human THBS2 protein (Catalog # 1635-T2-050; R&D, Minneapolis, MN, USA) for 24 h; 5-μm-thick heart tissue sections or cultured cells placed on 22 × 22 mm^2^ cover glass were incubated in blocking buffer (0.5% bovine serum albumin, 0.05% Tween-20, and PBS) for 1 h, followed by incubation with specific primary antibodies against EEA1 (Santa Cruz Biotechnology, Dallas, TX, USA), α-smooth muscle actin (SMA) (Bioss Antibodies Inc, Woburn, MA, USA), β-catenin (Cell Signaling Technology Inc, Danvers MA, USA), fibronectin (Santa Cruz Biotechnology), and vimentin (Santa Cruz Biotechnology) for 1 h. A fluorescence detection system (Ventana Medical Systems, Roche, Oro Valley, AZ, USA) was used, and cellular DNA was counterstained with DAPI (4′, 6-diamidino-2-phenylindole; Invitrogen). After washing four times, the sections were dried and mounted in VECTASHIELD^®^ mounting medium (Invitrogen) and then examined under an optical microscope or a confocal laser microscope (Leica) and Imaris 3D/4D image analysis software (Oxford Instruments, Concord, MA, USA).

### 2.7. Transmission Electron Microscopy (TEM)

The specimen preparation and analysis have been described previously [37]. In brief, HCMs and mouse cardiac tissue samples were fixed with 2.5% glutaraldehyde and 4% paraformaldehyde mixture in 0.01 M PBS for 2 h at 4 °C. After washing, cell pellets and the cardiac tissues were permeated in 2% agarose (in dH_2_O) and post-fixed with 1% osmium tetroxide in dH_2_O for 2 h. The samples were dehydrated in graded acetone, infiltrated, and then embedded in Epon LX112 (Ladd Research Industries, Vermont, USA)-acetone. Thin sections (70 nm) were sliced using a Leica microtome (Leica RM2165, Tokyo, Japan), stained with uranyl acetate and lead citrate, and examined using HITACHI HT-7700 transmission electron microscope (Tokyo, Japan) at an accelerating voltage of 80 kV. Images were captured using the built-in CCD image capture system of HITACHI HT7700.

### 2.8. Second-Harmonic Generation (SHG) Microscopy

To analyze the collagen deposition in the cardiac tissue sections without staining and de-waxing, SHG microscopy was performed. The Ti:Sapphire femtosecond laser (Tsunami, Spectra-Physics, Santa Clara, CA, USA) with a pulse width less than 100 fs and 80 MHz repetition rate was used. The wavelength was set to near-infrared (800 nm), and the power was adjusted to 22.4 mW on the specimen. The laser was tightly focused by a high numerical aperture (NA) objective lens (UPlanSAPo 20x/NA 0.75; Olympus, Ontario, Canada) and scanned using a pair of x-y galvanometers (6215H; Cambridge Technology, Cambridge, MA, USA) at pixel clock of 30 kHz to form 200 × 200 μm^2^ SHG images with 512 × 512-pixel resolution. The excited SHG signal was selected by a fluorescence bandpass filter (FF01-390/40-25; Semrock, Rochester, NY, USA) to remove the background autofluorescence. The collagen fibers distributed heterogeneously in cardiac tissue (10-μm-thick acute slices) could be naturally monitored and analyzed with sufficient resolution and high contrast. We performed the data analysis, including the SHG image processing and reconstruction, using ImageJ software (Bethesda, MD, USA).

### 2.9. MiRNA Extraction

MiRNA was extracted from extracellular vesicles abound in cell culture medium using EV-miR extraction kit (Topgen Biotech, Kaohsiung, Taiwan) following the manufacturer’s protocol and finally eluted in 20 μL low Tris-EDTA buffer (TE buffer) for each sample. The miRNA concentration was quantified by a Qubit microRNA assay kit using a Qubit fluorometer (Thermo Fisher Scientific, Waltham, MS, USA).

### 2.10. RNA Isolation

RNA fragments (<1000 nucleotides) were isolated from 200 μL serum samples using the LiqmiR miRNA extraction kit (Topgen Biotech). RNA isolation was performed according to the manufacturer’s instructions. Twenty femtomolar of cel-miR-39-3p (Topgen Biotech) was spiked in during lysis of the sample. RNA was eluted in 20 μL of nucleic acid stabilizing buffer (Topgen Biotech) and stored at −80 °C until further analysis.

### 2.11. MiRNA Reverse Transcription and Real-Time PCR

MiRNA (2 μL) was reverse transcribed to cDNA using the miRscript RT kit (Topgen Biotech) with specific miRNA stem-loop reverse-transcribed primers (Table S3) according to the manufacturer’s instructions. Diluted cDNA (1:5) was used for performing qPCR on StepOne Plus real-time PCR system (Applied Biosystems, Waltham, MA, USA) with ChamGE Probe qPCR Master Mix and TopQ miRNA probe qPCR assays for has-miR-29a-3p and cel-miR-39-3p (Topgen Biotech). Primer and probe sequences are listed in Table S3. Each sample was evaluated in triplicate. Nuclease-free H_2_O was used as a non-template negative control in each experiment. Amplification data were normalized to cel-miR-39-3p expression. Quantification of relative expression reported as arbitrary units (copies number) was performed using the 2^−∆∆Ct^ relative quantification method.

### 2.12. Nano Ultra-Performance Liquid Chromatographic System with Tandem Mass Spectrometry (nanoUPLC-MS/MS)

NanoUPLC-MS/MS analyses were performed on a nanoUPLC system (Waters, Milford, MA, USA) and an LTQ Qrbitrap Discovery hybrid Fourier transform mass spectrometer (Thermo Fisher Scientific) in positive ion mode as described previously [38]. Exosomal proteins were extracted (10 μg in 10 μL) and mixed with 100 μL acetone. After centrifugation for 10 min at 10,000× *g*, the supernatant was removed, and the protein residues were evaporated to dryness. Exosomal protein residues were re-dissolved with 16 μL NH_4_HCO_3_ aqueous solution (25 mM). After trypsin digestion, 2 µL of peptide solution was injected into nanoUPLC-MS/MS for protein identification. The MS/MS data were acquired in data-dependent mode, the raw data files were processed with Mascot Distiller software and then uploaded to the in-house Mascot server.

### 2.13. Proteomic Data Analysis

Proteomic data were analyzed using the using R software. Non-parametric Kruskal–Wallis and Dunn’s multiple tests were used to estimate the significant differences in protein expression data among the samples by FSA package (https://cran.r-project.org/web/packages/FSA/index.html, v0.8.31, 7 November 2021, R Package). Proteins with statistical significance (*p*-value < 0.05) were selected for gene ontology (GO) and Kyoto Encyclopedia of Genes and Genomes (KEGG) pathway enrichment analyses using Cluster Profiler (v3.18.1, 28 October 2020, R Package, School of Basic Medical Sciences, Southern Medical University, China) [39], and significant enrichment results (*p*-value < 0.05) were then visualized by ggplot2 (v 3.3.3, R Package). The number of expressed proteins from different samples was shown as a Venn diagram (v1.6.20, R Package) [40]. The expression levels of differentially expressed proteins among the samples were depicted as a heatmap drawn using pheatmap (http://CRAN.Rproject.org/package=pheatmap, v1.0.12, 4 January 2019, R Package).

### 2.14. Human MiR-29a-3p Mimic and Inhibitor Transfection

Human miR-29a-3p mimic (10 nM, MC12449, Life Technologies, Gaithersburg, MD, USA) and miR-29a-3p inhibitor (10 nΜ, MH12449, Life Technologies) were transfected to HCMs using Lipofectamine^®^ RNAiMAX Transfection Reagent (Catalog #13778-150, Thermo Fisher Scientific) according to the manufacturer’s instructions. After 48 h, the levels of miR-29a-3p targeted proteins were detected by western blot assay.

### 2.15. Western Blot Analysis

Western blot analyses were performed as described previously [37]. The protein concentrations of cell or tissue lysates were measured using the Lowry assay. Afterward, 40 μg protein samples were separated using 10% or 12% sodium dodecyl sulfate polyacrylamide gel electrophoresis (SDS PAGE, according to the molecular weight of the proteins of interest) and then electroblotted onto a nitrocellulose membrane. The membranes were blocked with BlockPRO blocking buffer (BP01-1L; Visual Protein, Taipei, Taiwan), immunoblotted with specific primary antibodies against THBS2 (1:1000, A8561; ABclonal, Woburn, MA, USA), HSP90 (1:500, sc-101494; Santa Cruz Biotechnology), transferrin (1:1000, GTX112729; Gene Tex, Irvine, CA, USA), CD 81 (1:500, GTX101766; Gene Tex), α-SMA (1:1000, bsm-52396R; Bioss Antibodies Inc, Woburn, MA, USA), β-catenin (1:1000, GTX632676; Gene Tex), Fibronetin (1:500, ab2413; Abcam, Cambridge, MA, USA), Vimentin (1:1000, SC-7557; Santa Cruz Biotechnology), and β-actin (1:1000, NB600-501; Novus Biologicals, Centennial, CO, USA), and then detected using horseradish peroxidase-conjugated secondary antibodies. The signals were visualized by chemiluminescence using an enhanced detection kit (ECL, TU-ECL03, TOOLS, Taipei, Taiwan).

### 2.16. MiR-29a-3p-Specific In Situ Hybridization (ISH) Assay

RNAscope™ HD detection reagents -red kit (Catalog #324500, Advanced Cell Diagnostics, Newark, CA, USA), RNAscope™ probe (Catalog # 895241-S1, Advance Cell Diagnostics), RNAscope^®^ target retrieval reagents (Catalog #322000, Advance Cell Diagnostics) and RNAscope^®^ wash buffer reagents (Catalog #310091, Advance Cell Diagnostics) were hybridized on 5 μm slides of mouse cardiac tissues following the ISH assay protocol. The images were acquired using Nikon’s Eclipse E600 research microscope (Nikon, Tokyo, Japan).

### 2.17. Immunohistochemistry (IHC) Staining

The sections from cardiac tissues were examined using the ultravision quanto detection system procedure (TL-125-QHD, Thermo Fisher Scientific). The cardiac tissue sections were incubated with anti-THBS2 (1:200, A8561, ABclonal), α-SMA (1:400, bsm-52396R, Bioss Antibodies Inc), β-catenin (1:400, GTX632676, Gene Tex), fibronectin (1:200, ab2413, Abcam) and vimentin (1:400, SC-7557, Santa Cruz Biotechnology) antibodies at 4 °C overnight. Then, the sections were treated with a biotinylated anti-mouse secondary antibody for 1 h. The slides were counterstained with Mayer’s hematoxylin for 2 min and then mounted. The images were acquired using Nikon’s Eclipse E600 research microscope (Nikon, Tokyo, Japan).

### 2.18. Enzyme-Linked Immunosorbent Assay (ELISA)

Human blood samples were collected, and the plasma was separated by centrifugation at 1500× *g* for 10 min at 4 °C. Plasma THBS2 levels were determined using human THBS2 ELISA Kit PicoKine™ (Catalog# EK0642; Boster Biological Technology, Pleasanton CA, USA) according to the manufacturer’s instructions.

### 2.19. Exosome Isolation

HCM cells were treated with or without MCT for 24 h. Cardiomyocyte exosomes were isolated using the Exosome Isolation Kit (Catalog # 4478359; Thermo Fisher Scientific) according to the manufacturer’s instructions. The cell culture medium was collected and centrifuged at 3000× *g* for 10 min at 4 °C. The medium was harvested and filtered and then centrifuged at 4500× *g* for 15 min at 4 °C. The medium was harvested and mixed with exosome isolation reagent (medium:reagent, 5:1) at 4 °C overnight. The medium was centrifuged at 10,000× *g* for 1.5 h at 4 °C. The pellets were collected and washed with 1× PBS and then centrifuged at 10,000× *g* for 1.5 h at 4 °C. The supernatant was removed, and then 60 exosome isolation lysis buffer (60 μL) was added to the pellet, which was stored at −70 °C for analysis.

### 2.20. Statistical Analysis

The data are presented as the median and interquartile range of each group and were analyzed using Mann–Whitney U-test or Kruskal–Wallis test. All statistical analyses were performed using the SigmaStat version 3.5 (Systat Software Inc., Chicago, IL, USA), and *p* < 0.05 was considered statistically significant.

## 3. Results

### 3.1. Successful Establishment and Evaluation of an MCT-Induced PAH and Cardiac Remodeling Mouse Model

**Figure 1:**
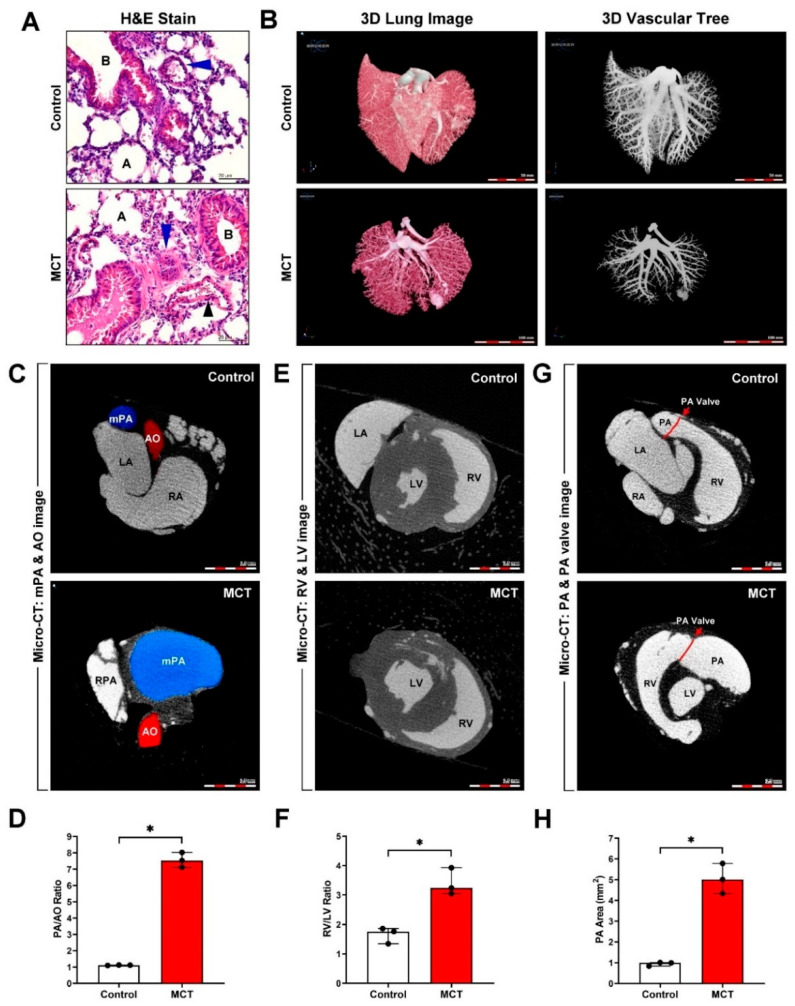
Monocrotaline (MCT)-induced pulmonary artery hypertension (PAH) in mice. C57BL/6 mice were treated with or without MCT (right side, 60 mg/kg; IP, once) for 30 days. (**A**) Hematoxylin and eosin (H&E) staining revealed a thicker pulmonary artery wall (blue arrows) following MCT-induced PAH. A, alveolar; B, bronchial; black arrows, vein. (**B**) The 3D lung image and vascular tree image were obtained by contrast-enhanced micro-computed tomography (MicroCT). (**C**,**D**) The mean pulmonary artery (mPA) and aortic artery (AO) image and PA/AO ratio; (**E**,**F**) left ventricle (LV) and right ventricle (RV) image and RV/LV ratio; (**G**,**H**) PA area was measured using MicroCT and Image J software. Data are representative of three independent experiments (*n* = 3), and values are expressed as median and interquartile range. Mann–Whitney U-test was used to compare two independent groups (* *p* < 0.05).

**Figure 2:**
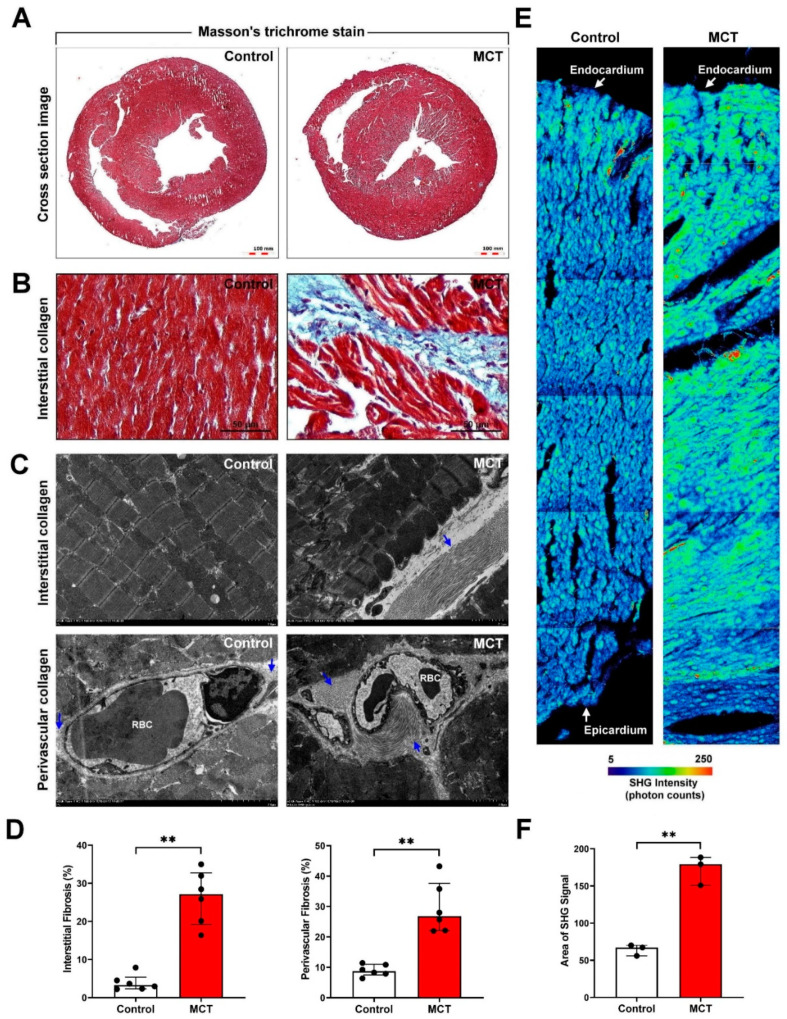
Myocardial fibrosis in MCT-induced PAH animal model. (**A**,**B**) Interstitial and perivascular collagen deposition in the transverse cardiac section was examined by Masson’s trichrome staining. Collagen fibers are indicated in blue. (**C**,**D**) Interstitial and perivascular collagen fiber accumulation in cardiac tissue was determined and quantified by transmission electron microscopy (TEM). Collagen fibers are indicated with blue arrows. (**E**,**F**) Cardiac collagen deposition and arrangement from the endocardium to epicardium were identified and quantified by second-harmonic generation (SHG) microscopy. Data are representative of three independent experiments (*n* = 3), and values are expressed as median and interquartile range. Mann–Whitney U-test was used to compare two independent groups (** *p* < 0.01).

We studied the regulatory mechanism of PAH-induced right heart failure and fibrosis using an MCT-induced PAH animal model. Murine lung tissue sections were examined by hematoxylin and eosin staining. It revealed increased medial wall thickness and fully vascularized vessels in the MCT-treated group compared with those in the control group (Figure 1A). High-resolution 3D microCT imaging was used to visualize the changes in pulmonary vascular morphometry. Significantly increased plexiform lesions and reduced pulmonary vascular connectivity density, vascular branching, and vascular lumen diameter were observed in the MCT-treated group compared with those in the control group (Figure 1B). The MCT-treated group showed significantly increased mean pulmonary artery (mPA) image and PA/aortic artery (AO) ratio (Figure 1C,D), RV image and RV/left ventricle (LV) ratio (Figure 1E,F), PA image, and PA area (Figure 1G and H). Thus, our experimental results indicated the successful establishment of an MCT-induced PAH mouse model. To further investigate whether PAH was caused by cardiac collagen deposition and fibrosis, the cardiac tissue sections were stained with Masson’s trichrome. A significant increase in perivascular and interstitial cardiac fibrosis (collagen, blue color) was observed in the MCT-treated group compared with that in the control group (Figure 2A,B). To determine the effects of MCT on collagen deposition in PAH-derived cardiac fibrosis, the interstitial and perivascular collagens were quantified by TEM analysis (collagen, blue arrow). Our results showed massive collagen accumulation in interstitial and perivascular cardiac tissues in the MCT-treated group compared with that in the control group (Figure 2C,D). Collagen accumulation in the endocardium to the pericardium was further determined and quantified by SHG microscopy. Our data showed that PAH induced significant collagen deposition and fibrosis in cardiac tissues in vivo (Figure 2E,F).

### 3.2. Cardiomyocyte-Derived Exosomes Exist in the Cardiac Microenvironment In Vitro and In Vivo

**Figure 3:**
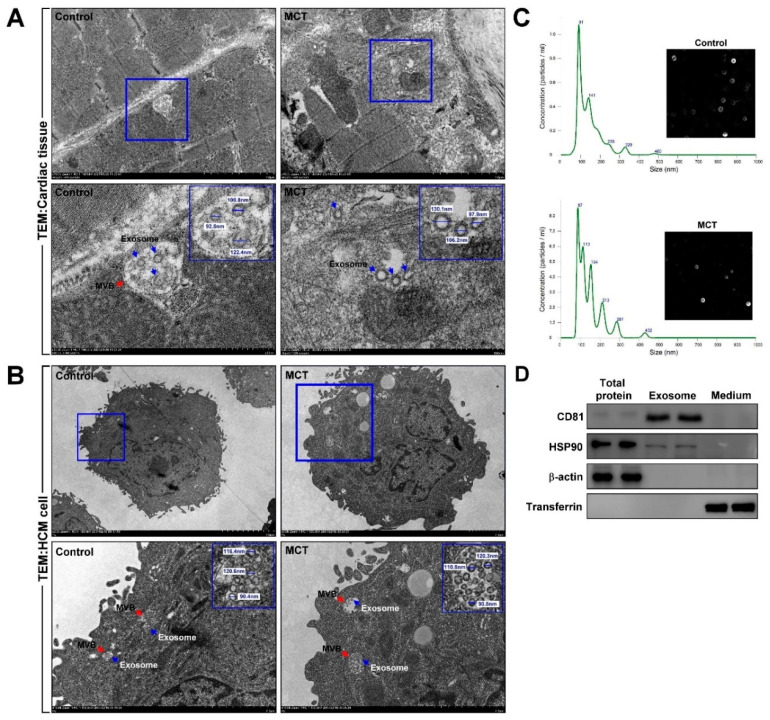
Cardiomyocyte-derived exosomes were detected in vitro and in vivo. (**A**,**B**) Multivesicular body (MVB) and exosome presence in myocardial tissue and human cardiomyocytes (HCM) were examined and quantified using TEM and Image J software. MVB, red arrow; exosome, blue arrow. (**C**) HCMs were treated with or without MCT for 24 h, and then the secreted exosomes were isolated from the culture medium. The exosome size was measured by nanoparticle tracking analysis. (**D**) The expression of exosomal proteins CD81 and HSP90 was examined by western blotting analysis. Data are representative of three independent experiments (*n* = 3).

To explore the existence of cardiomyocyte-derived exosomes in the cardiac microenvironment in vitro, multivesicular body (MVB) and exosome expression in cardiac tissues and HCMs were measured by TEM. Our data showed that MVB and exosomes were expressed in myocardial tissues and HCMs (Figure 3A,B). Next, to confirm the origin of the exosomes, HCMs were treated with or without MCT for 24 h, and then the cell culture medium was collected to isolate the exosomes. Exosome size was determined by nanoparticle tracking analysis. Our experimental results showed that MVB and exosomes were mostly expressed in cardiomyocytes rather than cardiac fibroblasts. The size of the exosomes was 90.4–130 nm (Figure 3C), which is consistent with the size range of 30–150 nm for exosomes [41]. Thus, our data showed the existence of exosomes in cultured cardiomyocytes as well as the murine cardiac microenvironment. To identify the HCM-secreted exosomes containing exosomes, the HCM-culture medium was collected, and exosomes were extracted. Forty micrograms of total proteins, exosomes, and culture medium were examined by western blotting. As shown in Figure 3D, exosome-specific markers, CD81 and HSP90, were detected in exosomal extracts, but not β-actin and transferrin.

### 3.3. Bioinformatics Analyses of MCT-Treated HCM-Derived Exosomes

**Figure 4:**
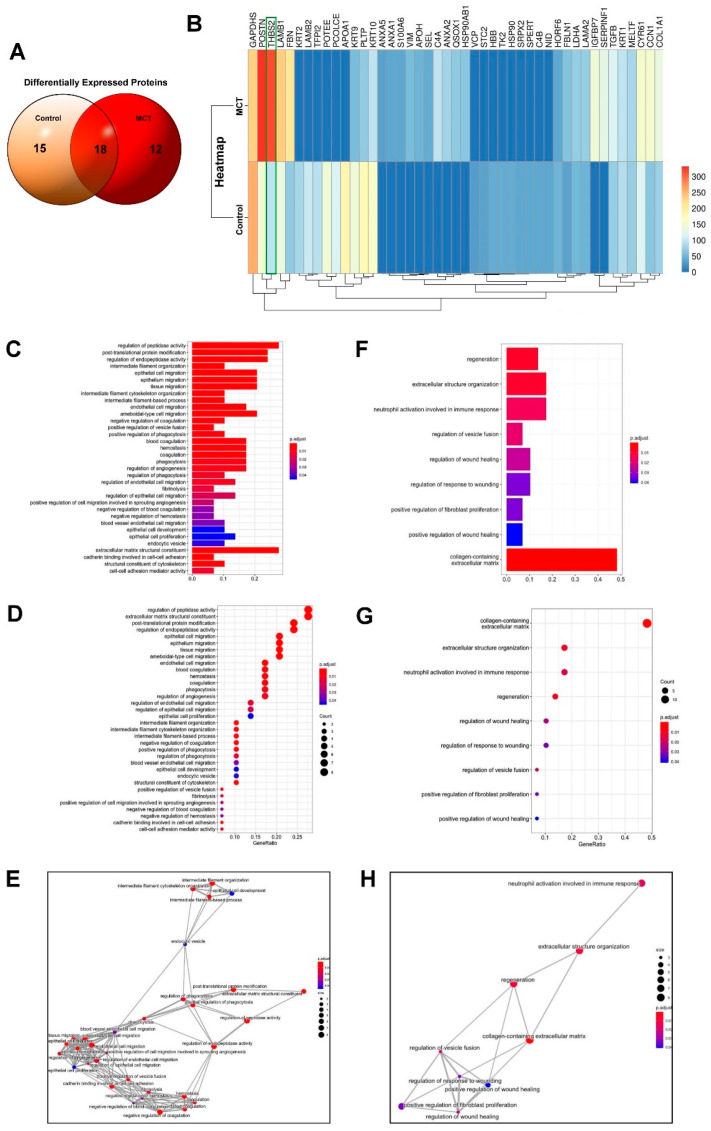
Proteomics profiling of HCM-derived exosomes. HCMs were treated with or without MCT for 24 h, and then the exosomal proteins were isolated and analyzed by high-resolution liquid chromatography-mass spectrometry (LC-MS/MS). (**A**) The intersection of the differentially expressed proteins between the control and MCT-treated samples was subjected to Venn diagram analysis. (**B**) The fold changes in gene expression between the experimental groups are illustrated with a heatmap. Genes are listed on the y-axis. (**C**–**E**) Functional annotation of differentially expressed genes in MCT-treated HCMs were analyzed using gene ontology (GO) barplot, dotplot, and emapplot analyses. (**F**–**H**) Extracellular structure organization and regeneration-associated genes identified using these analyses of differentially expressed genes in MCT-treated HCMs. Data are representative of three independent experiments (*n* = 3).

We further analyzed the changes in HCM-derived exosomal proteins under MCT treatment using proteomics profiling. HCMs were treated with or without MCT for 24 h, and then exosomal proteins were isolated and analyzed using nanoUPLC-MS/MS. The different exosomal proteins expressed between the control and MCT-treated groups were analyzed using a Venn diagram. Exosomal proteins specifically expressed in the control group were APOA1, POTEE, PCOLCE, LAMB2, TFPI2, KRT2, HBB, TK2, HSP90, SRPX2, C4B, SPERT, NID, STC2, and VCP, whereas those exclusively expressed in the MCT-treated group were IGFBP7, SERPINF1, C4A, ANXA2, QSOX1, HSP90AB1, VIM, APOH, SEL, ANXA1, S100A6, and ANXA5. Moreover, GAPDHS, POSTN, THBS2, LAMB1, PLTP, FBN, CYR61, KRTCN1, KRT9, COL1A1, TGFB, KRT1, MELTF, LDHA, LAMA2, FBLN1, and HORF6 were commonly expressed in both groups (Figure 4A). The results of heatmap analysis indicated that MCT upregulated the expression of POSTN, THBS2, FBN, CCN1, IGFBP7, SERPINF1, C4A, ANXA2, QSOX1, HSP90AB1, VIM, APOH, SEL, ANXA1, S100A6, and ANXA5 but downregulated that of PLTP, KRT10, KRT9, APOA1, POTEE, PCOLCE, LAMB2, TFPI2, KRT2, HORF6, HBB, TK2, HSP90, SRPX2, C4B, SPERT, NID, STC2, and VCP. Notably, the expression of THBS2 was significantly increased in the MCT-treated group compared with that in the control group (3.326-fold, *p* = 0.00013) (Figure 4B). We further analyzed the uniquely expressed exosomal proteins in the MCT-treated group using the GO analysis, which grouped the results into biological process, cellular component, and molecular function. The identified proteins were significantly enriched in “cell migration” (Figure 4C–E) and “fibrosis” (Figure 4F–H) after MCT treatment. Furthermore, the GO analysis of the common exosomal proteins between the groups showed that most proteins in the cellular component were enriched in “regulation of cell migration”, “blood coagulation”, “angiogenesis” and “cytoskeleton organization” in the MCT-treated group. Additionally, MCT exosomal proteins were also enriched in “collagen-containing ECM”, “fibroblast proliferation”, “wound healing” and “regeneration”. Moreover, in the KEGG pathway analysis, the commonly expressed exosomal proteins were significantly enriched in “ECM structural constituent” and “collagen-containing ECM” (Figure 4D,G). According to the results of the heatmap analysis, THBS2 is involved in regulating ECM structural constituent and collagen-containing ECM, which were enriched in exosomes isolated from the MCT-treated group.

### 3.4. MiR-29a-3p Modulates THBS2 Expression In Vitro and In Vivo

**Figure 5:**
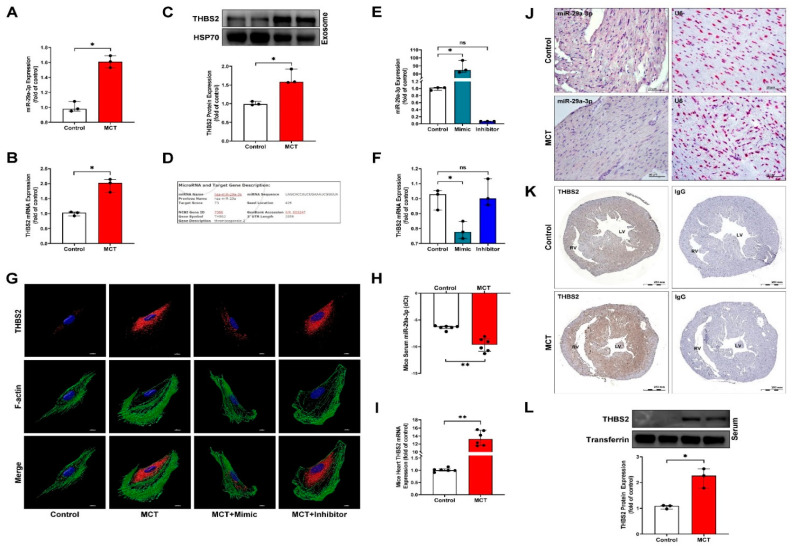
miR-29a-3p regulates THBS2 expression in vitro and in vivo. (**A**,**B**) MCT-induced expression of miR-29a-3p and exosomal THBS2 mRNA, determined by real-time PCR. (**C**) THBS2 expression in MCT-treated HCM-secreted exosomes determined by western blotting. HSP70 was used as the internal control for exosomal proteins. (**D**) Alignment of the miR-29a-3p with the THBS2 3′-untranslated region, showing the putative binding sites. (**E**) Real-time PCR analysis of miR-29a-3p expression in HCMs after transfection with an miR-29a-3p mimic or inhibitor. Data are representative of three independent experiments (*n* = 3), and values are expressed as median and interquartile range. Mann–Whitney U-test was used to compare two independent groups (* *p* < 0.05). (**F**,**G**) After transfection, THBS2 expression was analyzed by real-time PCR and immunocytochemical staining. Data are representative of three independent experiments (*n* = 3), and values are expressed as median and interquartile range. Kruskal–Wallis test was used to compare two independent groups (* *p* < 0.05). (**H**,**I**) MCT-induced expression of miR-29a-3p and THBS2 mRNA, determined by real-time PCR. Mann–Whitney U-test was used to compare two independent groups (** *p* < 0.01). (**J**) In situ hybridization (ISH) of miR-29a-3p expression (pink color) in myocardial tissues of MCT-induced PAH mice. U6, miRNA internal control. (**K**,**L**) THBS2 expression in myocardial tissues and circulating serum of MCT-induced PAH mice were examined by immunohistochemistry and western blotting, respectively. Transferrin was used as the internal control. Data are representative of three independent experiments (*n* = 3), and values are expressed as median and interquartile range. Mann–Whitney U-test was used to compare two independent groups (* *p* < 0.05 and ** *p* < 0.01).

To estimate the presence of miRNA-targeted sites in THBS2, we used the microRNA.org website (http://www.microrna.org/microrna/home.do, https://bioweb.pasteur.fr/packages/pack@miRanda@3.3a, v3.3a, 10 March 2020) and found that THBS2 (NM_003247) contains potential binding sites for miR-29a-3p (seed location 426). Compared with the control group, MCT treatment reduced the expression of miR-29a-3p in HCMs, while increasing the expression of THBS2 in cardiomyocyte-derived exosomes and the cardiomyocyte culture medium (Figure 5A–F). In addition, overexpression of miR-29a-3 significantly reduced THBS2 expression by binding to its 3′-untranslated region, whereas silencing miR-29a-3p expression increased THBS2 expression in HCMs (Figure 5G). In PAH model mice, the miR-29a-3p level was examined by qPCR and in situ hybridization analyses (Figure 5H,J); THBS2 mRNA and protein in myocardial tissue were measured by qPCR, immunohistochemical staining, and western blotting (Figure 5I,K,L). The results indicated that the miR-29a-3p level was significantly reduced, and the expression of THBS2 in tissues was increased in the MCT-induced PAH animal model.

### 3.5. Cardiomyocyte-Derived Exosomal THBS2 Regulates Activation and Transformation of Cardiac Fibroblasts and Induces Cardiac Fibrosis

**Figure 6:**
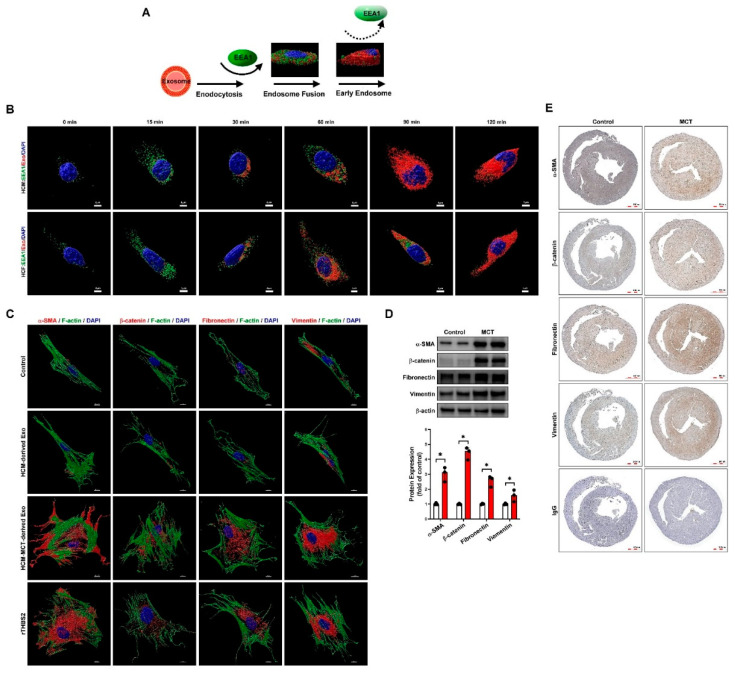
Cardiomyocyte-derived exosomes regulate cardiac fibroblast to myofibroblast transformation. (**A**) Schematic diagram for studying the effects of human cardiomyocyte (HCM)-derived exosome uptake by human cardiac fibroblasts (HCF) using early endosome antigen 1(EEA1) staining. (**B**) Immunocytochemistry and confocal microscopy identified the time-dependent intake and regulation of HCM-derived exosomes by EEA1 in HCFs. Nuclear DNA, exosomes, and EEA1 were labeled with 4′,6-diamidino-2-phenylindole (DAPI, blue), Dil dye (1,1′-dioctadecyl-3,3,3′,3′-tetramethylindocarbocyanine, red), and GFP fluorescence dye (green), respectively. Data are representative of three independent experiments (*n* = 3). (**C**) HCFs were treated with or without HCM-derived exosomes, HCM+MCT-derived exosomes, or rTHBS2 (THBS2 recombinant protein, 1 μg/mL) for 48 h to assess cardiomyocyte-derived exosomal THBS2-mediated fibroblast to myofibroblast transformation. Expression of the myofibroblast marker proteins, α-SMA, β-catenin, fibronectin, and vimentin was examined by immunocytochemistry and confocal microscopy with Imaris software for confocal image 3D reconstruction. (**D**,**E**) Myofibroblast marker protein expression in myocardial tissues of MCT-induced PAH mice was examined by western blotting and immunohistochemistry. Data are representative of three independent experiments (*n* = 3), and values are expressed as median and interquartile range. Mann–Whitney U-test was used to compare two independent groups (* *p* < 0.05).

To investigate the role of cardiomyocyte-derived exosomes in PAH-induced cardiac fibrosis, HCMs were treated with or without MCT and labeled with Dil fluorescence (red fluorescence) for various periods (0–120 min). The expression of labeled exosomes and EEA1 was detected by confocal microscopy (Figure 6A,B). We found that cardiomyocyte-derived exosomes were taken up by HCFs in a time-dependent manner. In addition, to evaluate whether fibroblast to myofibroblast transformation is regulated by HCM-derived exosomal THBS2, the expression of key myofibroblast marker proteins was examined by western blotting and immunocytochemistry. The results indicated that MCT-treated HCM-derived exosomes and THBS2 recombined protein treatment significantly increased fibroblast to myofibroblast transformation (Figure 6C). The PAH animal model also confirmed that PAH-induced myofibroblast marker proteins were highly expressed in the myocardial tissue (Figure 6D,E).

### 3.6. Circulating MiR-29a-3p and THBS2 Levels Are Prognostic Markers for Disease Severity of PAH

**Figure 7:**
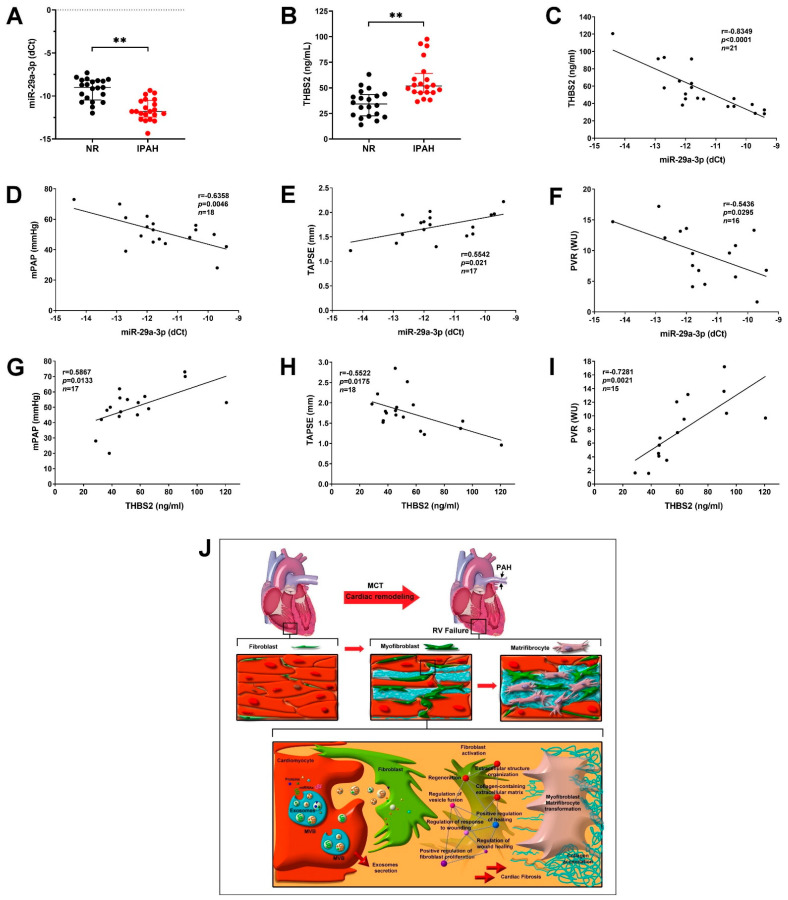
MiR-29a-3p/THBS2 axis regulates the progression of pulmonary artery hypertension (PAH). (**A**) Exosomal miR-29a-3p level in the serum of healthy controls (NR) and patients with PAH was determined by real-time PCR. (**B**) THBS2 expression in the serum was determined by ELISA. Data are expressed as median and interquartile range. Mann–Whitney U-test was used to compare two independent groups (** *p* < 0.01). (**C**) Correlation coefficient between the miR-29a-3p and THBS2 expression levels. R, Pearson’s correlation coefficient. (**D**–**F**) The correlation of miR-29a-3p with the mean pulmonary artery pressure (mPAP), tricuspid annular plane systolic excursion (TAPSE), and pulmonary vascular resistance (PVR) in patients with PAH. (**G**–**I**) The correlation of the serum THBS2 level with mPAP, TAPSE, and PVR in patients with PAH. (**J**) Suggested mechanism underlying PAH-induced cardiac fibrosis mediated by exosomal miR-29a-3p and THBS2. Serum miR-29a-3p and THBS2 (secreted by cardiomyocytes) might increase cardiomyocyte hypertrophy and promote cardiac fibroblast to myofibroblast transformation, enhance collagen production/deposition, and augment cardiac fibrosis.

Next, we examined the exosomal miR-29a-3p and THBS2 expression levels in the serum of patients with PAH and assessed the correlation between the donor and IPAH groups. The miR-29a-3p level in the PAH group was significantly lower than that in the donor group (Figure 7A), whereas THBS2 expression was significantly higher in the PAH group (Figure 7B). Furthermore, a negative correlation between the miR-29a-3p and THBS2 (Figure 7C) levels was detected in patients with PAH. To elucidate whether the expression of miR-29a-3p and THBS2 may be functional predictors of RV function prognosis and PAH outcome, echocardiographic parameter (TAPSE) and Swan-Ganz parameters (mPAP and pulmonary vascular resistance (PVR)) were used for analysis. miR-29a-3p negatively correlated with mPAP and PVR and positively correlated with TAPSE; THBS2 showed a positive correlation with mPAP and PVR and a negative correlation with TAPSE (Figure 7D–I). These results indicated the regulatory effect of the miR-29a-3p/THBS2 axis in PAH-induced cardiac fibrosis and were consistent with the results of our in vitro experiments, PAH mouse model analysis, and clinical evidence. Thus, PAH regulated the secretion of miR-29a-3p/THBS2 from cardiomyocytes and subsequent uptake by cardiac fibroblasts. The miR-29a-3p/THBS2 axis mediated the cardiac fibroblast to myofibroblast transition through ECM structural constituents and collagen-containing ECM pathway, thereby increasing PAH-induced cardiac fibrosis (Figure 7J).

## 4. Discussion

In the present study, we demonstrated that MCT-induced cardiomyocyte-derived exosomes are rich in THBS2, and these could be taken up by cardiac fibroblasts, resulting in their activation and thereby promoting cardiac fibrosis. Our findings indicate that serum miR-29a-3p, THBS2, and miR-29a-3p/THBS2 ratio exhibit diagnostic and prognostic values in patients with IPAH; in particular, their direct correlations with clinical parameters, such as mPAP, PVR, and TASPE, are promising in PAH. Additionally, our experimental results showed that miR-29a-3p targets THBS2, leading to decreased cardiac fibroblast activation and profibrotic signaling pathway activation. Consistently, we observed decreased expression of miR-29a-3p and increased expression of THBS2 in the serum of the PAH model mice as well as patients with PAH. Our experimental results indicated that HCM-derived exosomal THBS2 acts as a paracrine signaling mediator and is involved in the induction of cardiac fibroblast activation and myofibroblast transformation. Additionally, the bioinformatic analysis revealed that exosomes containing THBS2 secreted by PAH-cardiomyocytes regulate the signaling pathways associated with the collagen-containing ECM, fibroblast proliferation, wound healing, and regeneration. A previous study reported that THBS2 is highly expressed in hypertensive heart failure, and serum THBS2 levels were associated with the New York Heart Association (NYHA) functional class [42]. Consistently, we found significantly higher circulating serum THBS2 levels in patients with PAH than in healthy controls. Furthermore, a high serum THBS2 level is reportedly associated with anemia [43], renal dysfunction [44], and heart failure severity [45]. Increasing the level of circulating THBS2 is a compensatory response to cardiac overloads, such as an increase in B-type natriuretic peptides (BNP) [14]. Moreover, the increased secretion of THBS2 into the circulation is a compensatory mechanism in heart failure conditions. Our experimental results further confirmed that THBS2 could be used to predict the exacerbation of pulmonary hypertension and right heart failure. The THBS2 level significantly correlated with the PVR, TASPE, and mPAP changes in the present study. Currently, THBS2 is considered a potentially useful predictor of future adverse cardiovascular events in patients with cardiac fibrosis [10,46]; however, its role in PAH has not been clarified to date. The present study is the first to explore the correlation between THBS2 and PAH and demonstrate that THBS2 is an effective predictor of PAH prognosis.

Circulating miRNA levels are considerably correlated with their corresponding levels in the cardiac microenvironment [47]. Circulating miRNAs reflect the changes occurring in the myocardial microenvironment due to PAH. It has been recognized that even insignificant small changes in the expression of genes associated with specific pathways may exert significant biological effects [48]. Therefore, circulating miRNAs are a useful diagnostic and prognostic biomarker of PAH [49,50]. Previous studies have reported the association of circulating miRNAs, including miR-27b and miR-451 [51], miR-125a-5p [52], miR-509-3p [53], miR-206 [54], miR-483 [55], miR-211 [56], miR-143 [57], miR-596 [58], and miR-208-Mef2 [59] axis with the prognosis of PAH. The miR-29 family members have been reported as anti-fibrotic miRNAs [48]. miR-29 overexpression targets a broad spectrum of fibrosis-related genes, and cardiomyocyte-derived miR-29 promotes pathological remodeling of hypertrophy and fibrosis by activating Wnt signaling [33]. Although miR-29 has been reported to be a potent therapeutic miRNA for treating pulmonary fibrosis [60], the performance and influence of circulating serum miR-29a-3p in PAH have not been explored. We found decreased miR-29a-3p levels in MCT-treated HCMs, serum, and cardiac tissues of MCT-induced PAH model mice, as well as serum of patients with IPAH. In addition, miR-29a-3p was strongly correlated with PAH diagnosis and prognosis parameters, including PVR, mPAP, and TASPE.

Another key finding of the present study is the miR-29a-3p-induced regulation of THBS2. THBS2 is a pro-fibrotic, anti-angiogenic matricellular protein and an attractive target gene shown to be modulated by different miRNAs, including miR-221-3p [61], miR-9 [62], and miR-29a-3p [46,63]. The activation of pulmonary adventitial fibroblasts plays a crucial role in pulmonary vascular remodeling in hypoxic pulmonary hypertension. An miR-29a-3p mimic was found to inhibit pulmonary adventitial fibroblast activation, significantly decrease pulmonary artery pressure and right ventricle hypertrophy index, and ameliorate pulmonary vascular remodeling in hypoxic pulmonary hypertension rats [27]. Our in vitro results showed that the overexpression of miR-29a-3p inhibited cardiac fibroblast activation and fibroblast to myofibroblast transformation and significantly decreased the expression of profibrotic markers.

This study had a few limitations. The number of patients with IPAH was small, which limited the assessment of miR-29a-3p/THBS2 correlation with cardiac output, RV pressure, and pulmonary capillary wedge pressure. We did not perform Swan-Ganz implantation in the PAH model mice to detect parameters such as cardiac output, RV pressure, pulmonary capillary wedge pressure, and pulmonary artery pressure. These parameters could help verify the accuracy of miR-29a-3p/THBS2 in assessing the prognosis of pulmonary hypertension.

## 5. Conclusions

In summary, our study demonstrated that PAH reduces the circulating miR-29a-3p level and increases cardiomyocyte-derived exosomal THBS2 secretion to regulate cardiac fibroblast to myofibroblast transformation and PAH-related cardiac fibrosis progression. Our study findings indicate that the miR-29a-3p/THBS2 axis is a potential target for PAH, broadening the understanding of miR-29a-3p and cardiomyocyte-derived exosome-mediated fibrotic response. Collectively, these results suggest that miR-29a-3p/THBS2 could be used to evaluate the clinical prognosis of PAH.

## Data Availability

Original data will be available upon request.

## Supplementary Materials

The following are available online at https://www.mdpi.com/article/10.3390/ijms221910574/s1, Table S1: Clinical characteristics of IPAH patients. Table S2: Clinical characteristics of healthy donors. Table S3: Primer and probe sequences.
