# Supplementary material for: miR-29a-3p/THBS2 Axis Regulates PAH-Induced Cardiac Fibrosis
Source: Int J Mol Sci. 2021 Sep 30;22(19):10574. doi: 10.3390/ijms221910574 (PMC8509017; doi:10.3390/ijms221910574)
Supplement: Supplementary file 1 [file ijms-22-10574-s001.zip › ijms-1368297-supplementary.pdf]

International Journal of *Molecular Sciences*

MDPI

# miR-29a-3p/THBS2 axis regulates PAH-induced cardiac fibrosis

## Supplementary Materials:

**Table S1. Clinical characteristics of IPAH patients.**

| Diagnosis | Clinical Characteristics | | | | | Echocardiography | | | | | | Right Heart Catheterization | | | | |
|---|---|---|---|---|---|---|---|---|---|---|---|---|---|---|---|---|
| | Number | Sex | Age (y) | H/T | DM | RA/RV Dilated | D-shape LV | EF (%) | RV'S (cm/s) | PA (mmHg) | TAPSE | RA (mmHg) | m PAP (mmHg) | PCWP (mmHg) | C.O (L/min) | PVR (WU) |
| IPAH | 1 | M | 84 | Y | N | RV | N | 77.2 | 17.6 | 61 | 2.52 | 4 | 20 | 14 | 5.41 | 1.1 |
| | 2 | F | 49 | N | N | RA/RV | Y | 71.5 | 9.16 | 76 | 0.96 | 3 | 49 | 3 | 3.5 | 13.14 |
| | 3 | F | 55 | N | N | RA/RV | Y | 79.3 | 12.6 | 189 | 1.95 | 15 | 61 | 17 | 3.48 | 12.06 |
| | 4 | M | 37 | N | N | RA/RV | Y | 75.7 | 10 | 57 | 1.3 | 9 | 47 | 10 | 5.48 | 6.75 |
| | 5 | F | 52 | N | N | RA/RV | Y | 78.5 | 9.36 | 177 | 1.22 | 14 | 53 | 21 | 3.3 | 9.69 |
| | 6 | F | 28 | N | N | RV | Y | 64.1 | 11 | 72 | 1.65 | 8 | 55 | 10 | 9.73 | 3.49 |
| | 7 | F | 36 | N | N | RA/RV | Y | 77.8 | 10.1 | 79 | 1.65 | 6 | 45 | 10 | 4.48 | 7.8 |
| | 8 | F | 36 | N | N | RA/RV | Y | 65.3 | 11.5 | 87 | 2.22 | 9 | 42 | 11 | 4.57 | 6.78 |
| | 9 | F | 40 | N | N | RA/RV | Y | 62.4 | 12.8 | 36 | 1.88 | 7 | 40 | 11 | 7.25 | 4 |
| | 10 | F | 40 | N | N | RA/RV | Y | 72.9 | 11.5 | 44 | 1.95 | 10 | 45 | 13 | 7.33 | 4.1 |
| | 11 | M | 56 | N | N | RA/RV | Y | 78.5 | 14.2 | 64 | 1.7 | 12 | 56 | 14 | 7.27 | 5.7 |
| | 12 | F | 76 | Y | Y | Mild RA | N | 69.3 | 12.1 | 101 | 2.02 | 8 | 53 | 14 | 4.5 | 7.55 |
| | 13 | F | 32 | N | N | RA/RV | Y | 63.2 | 11.5 | 103 | 1.37 | 6 | 70 | 12 | 3.37 | 17.2 |
| | 14 | M | 32 | N | N | RA/RV | Y | 71.5 | 15.2 | 118 | 1.81 | 19 | 62 | 13 | 3.58 | 13.6 |
| | 15 | F | 34 | N | N | N | N | 73.8 | 11.7 | 57 | 1.79 | 12 | 20 | 12 | 5.09 | 1.57 |
| | 16 | F | 50 | N | N | RV | Y | 82.49 | 15.5 | 83 | 2.85 | 12 | 44 | 15 | 6.46 | 4.49 |
| | 17 | F | 48 | N | N | RA/RV | Y | 73.2 | 9.46 | 96 | 1.75 | 8 | 50 | 12 | 2.84 | 13.3 |
| | 18 | F | 73 | N | Y | Mild RV | N | 69.9 | 11.2 | 61 | 1.56 | 13 | 73 | 15 | 5.33 | 10.8 |
| | 19 | F | 72 | N | Y | LA/RA/RV | Y | 71.28 | 10.7 | 112 | 1.52 | 10 | 48 | 9 | 2.65 | 14.7 |
| | 20 | M | 52 | N | Y | RA/RV | N | 75.8 | 16.4 | 60 | 1.97 | 7 | 28 | 14 | 8.57 | 1.63 |
| | 21 | F | 60 | Y | N | RA/RV | Y | 79 | 12 | 77 | 1.89 | 20 | 57 | 14 | 4.52 | 9.51 |

Abbreviations: H/T, hypertension; DM, diabetes; RA, right atrial; RV, ventricular; PA, estimated systolic pulmonary pressure; TAPSE, tricuspid annular plane systolic excursion; EF, ejection fraction; RV-S′, longitudinal velocity of the tricuspid annulus (measure by tissue Doppler); mPAP, mean pulmonary artery pressure; PVR, pulmonary vascular resistance; C.O, cardiac output; PCWP, pulmonary capillary wedge pressure; D-shape LV, D-shaped left ventricle (Flattening of the interventricular septum detected during echocardiographic examination is called D-shaped left ventricle)

**Table S2. Clinical characteristics of healthy donors.**

| Clinical characteristics | | | | | Echocardiography | | |
|---|---|---|---|---|---|---|---|
| **Number** | **Sex** | **Age (y)** | **H/T** | **DM** | **EF (%)** | **RV-S′ (cm/s)** | **PA (mmHg)** |
| 1 | F | 30 | N | N | 69.6 | N/A | 27 |
| 2 | M | 44 | N | N | 56.2 | N/A | <20 |
| 3 | F | 32 | N | N | 78 | N/A | 33 |
| 4 | M | 43 | N | N | 66.8 | N/A | <20 |
| 5 | F | 37 | N | N | 80.5 | N/A | <20 |
| 6 | M | 38 | N | N | 59.5 | N/A | <20 |
| 7 | M | 27 | N | N | 70.7 | N/A | 24 |
| 8 | M | 38 | N | N | 71.9 | N/A | 28 |
| 9 | F | 22 | N | N | 53 | N/A | 25 |
| 10 | F | 33 | N | N | 75.4 | N/A | <20 |
| 11 | F | 36 | N | N | 70.6 | N/A | <20 |
| 12 | F | 44 | N | N | 53.9 | N/A | <20 |
| 13 | F | 37 | N | N | 64.8 | N/A | 28 |
| 14 | F | 45 | N | N | 71.9 | N/A | <20 |
| 15 | M | 25 | N | N | 73 | N/A | <20 |
| 16 | M | 34 | N | N | 70.4 | N/A | 28 |
| 17 | F | 31 | N | N | 65 | N/A | 26 |
| 18 | F | 45 | N | N | 63 | N/A | 22 |
| 19 | M | 29 | N | N | 68.2 | N/A | 26 |
| 20 | F | 38 | N | N | 73.8 | N/A | 28 |
| 21 | M | 44 | N | N | 58.6 | N/A | 29 |

Abbreviations: H/T, hypertension; DM, diabetes; EF, ejection fraction; RV-S′, longitudinal velocity of the tricuspid annulus (measure by tissue Doppler); PA, estimated systolic pulmonary pressure.

**Table S3. Primer and probe sequences.**

| Assay Name | 5′ labeled | Sequence (5′–3′) | 3′ labeled |
| --- | --- | --- | --- |
| hsa-miR-29a-3p reverse transcribed primer | | GTCGTATCCAGTGCAGGGTCCGAGGTATTCGCACTG GATACGACTAACCG | |
| hsa-miR-29a-3p-F | ---- | CGCGTAGCACCATCTGAAAT | ---- |
| hsa-miR-29a-3p-R | ---- | AGTGCAGGGTCCGAGGTATT | ---- |
| hsa-miR-29a-3p-P | FAM | ACTAACCGATTTCAG | MGB |
| cel-miR-29a-3p reverse transcribed primer | | GTCGTATCCAGTGCAGGGTCCGAGGTATTCGCACTG GATACGACCAAGCT | |
| cel-miR-39-3p-F | ---- | CGCGTCACCGGGTGTAAA | ---- |
| cel-miR-39-3p-3p-R | ---- | AGTGCAGGGTCCGAGGTATT | ---- |
| cel-miR-39-3p-P | FAM | ACGACCAAGCTGATTTACAC | MGB |

FAM, 6-carboxyfluorescein; MGB, minor groove binder
